# Machine Learning-Based Prediction of Rule Violations for Drug-Likeness Assessment in Peptide Molecules Using Random Forest Models

**DOI:** 10.3390/ijms26178407

**Published:** 2025-08-29

**Authors:** Momchil Lambev, Dimana Dimitrova, Silviya Mihaylova

**Affiliations:** Medical College, Medical University of Varna, 84 Tzar Osvoboditel Str., 9002 Varna, Bulgariasilviya.mihaylova@mu-varna.bg (S.M.)

**Keywords:** Random Forest, machine learning, drug-likeness, peptides, Lipinski Rule of Five, beyond Rule of Five, Muegge’s rule, oral bioavailability

## Abstract

Peptide therapeutics often fall outside classical small-molecule heuristics, such as Lipinski’s Rule of Five (Ro5), motivating the development of adapted filters and data-driven approaches to early drug-likeness assessment. We curated >300 k drug (small and peptide) and non-drug molecules from PubChem, extracted key molecular descriptors with RDKit, and generated three rule-violation counters for Ro5, the peptide-oriented beyond-Ro5 (bRo5) extension, and Muegge’s criteria. Random Forest (RF) classifier and regressor models (with 10, 20, and 30 trees) were trained and evaluated. Predictions for 26 peptide test molecules were compared with those from SwissADME, Molinspiration, and manual calculations. Model metrics were uniformly high (Ro5 accuracy/precision/recall = 1.0; Muegge ≈ 0.99), indicating effective learning. Ro5 violation counts matched reference values for 23/26 peptides; the remaining cases differed by +1 violation, reflecting larger structures and platform limits. bRo5 predictions showed near-complete agreement with manual values; minor discrepancies occurred in isolated peptides. Muegge’s predictions were internally consistent but tended to underestimate SwissADME by ~1 violation in several molecules. Four peptides (ML13–16) satisfied bRo5 boundaries; three also fully met Ro5. RF models thus provide fast and reliable in silico filters for peptide drug-likeness and can support the prioritisation of orally developable candidates.

## 1. Introduction

High-throughput screening and combinatorial chemistry represent essential components in the drug discovery process [1,2,3]. The increasing capabilities for compound synthesis and screening necessitate the development of methods for evaluating their potential to be incorporated into the drug development pipeline [4,5,6,7]. This, in turn, requires the establishment of databases that significantly enhance the likelihood of identifying compounds suitable for further investigation. Molecules exhibiting such characteristics are referred to as “drug-like”. The term also implies the feasibility of synthesising the compound and its analogues. Moreover, drug-like compounds are expected to comply with ADME (absorption, distribution, metabolism, and excretion) and toxicological profiles [8,9].

### 1.1. Drug-Likeness and the Evolution of Structural Filters for Oral Bioavailability

Lipinski and colleagues proposed what is arguably the most widely recognised rule in drug design, aiming to identify compounds with favourable absorption characteristics based on their physicochemical properties [10]. Commonly referred to as the Lipinski Rule of Five (Ro5), this heuristic approach, formulated in 1997, evaluates a compound’s drug-likeness by assessing its likelihood of achieving sufficient oral bioavailability. The Ro5 is grounded in pharmacokinetic principles, encompassing absorption, distribution, metabolism, and excretion, and establishes the following threshold values for key molecular parameters: logP between 0 and 5; molecular weight (Mw) ≤ 500 Da; hydrogen bond acceptors (HBA, i.e., nO, nN) ≤ 10; hydrogen bond donors (HBD, i.e., nOH, nNH) ≤ 5. According to the rule, a compound is more likely to exhibit good oral bioavailability if it does not violate more than one of these parameters. However, while these guidelines provide a helpful framework, they are not definitive predictors of drug suitability. Numerous factors beyond these parameters influence a molecule’s pharmacokinetics and pharmacodynamics, necessitating a broader evaluation strategy.

Notably, peptide-based drugs often fall outside the traditional Ro5 criteria. These molecules typically possess high molecular weights, significant hydrophilicity, limited stability, and heterogeneous physicochemical profiles, posing challenges for oral delivery. Consequently, the Ro5 has been adapted to reflect the unique characteristics of peptides better. This revised framework, known as beyond Rule of Five (bRo5), proposes the following extended thresholds: Mw ≤ 1000 Da, −2 ≤ cLogP ≤ 10, HBD ≤ 6, HBA ≤ 15, polar surface area (PSA) ≤ 250 Å^2^, and number of rotatable bonds (NRotB) ≤ 20. Doak et al. [11] reported that 25 orally approved peptide drugs exhibit molecular weights ranging from 700 to 929 Da, with a mean of approximately 815 Da, illustrating the increasing relevance of bRo5-compliant compounds in drug development.

To refine the ability to discriminate between drug-like and non-drug-like molecules using structural features, Muegge et al. [12] expanded upon Lipinski’s model by integrating topological criteria proposed initially by Bemis and Murcko [13]. These include additional structural descriptors and requirements for elemental composition. Specifically, Muegge’s criteria emphasise the presence of carbon or heteroatoms such as (N, O, S, H, P, Si, Cl, Br, F, I) [12], further enhancing the predictive power of computational drug-likeness filters.

Collectively, these evolving frameworks underscore the need for adaptable and nuanced screening methodologies, particularly as drug discovery increasingly encompasses structurally diverse and complex therapeutic modalities such as peptides.

### 1.2. Random Forest Algorithm

**Figure 1:**
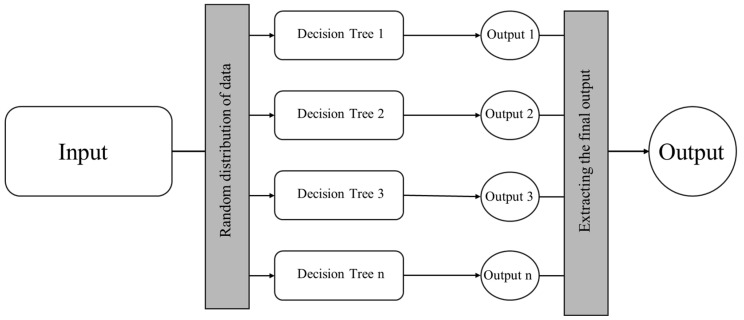
Random forests workflow (source: authors).

Traditional algorithms commonly used in machine learning (ML) often suffer from low accuracy and are prone to overfitting. To address these limitations, ensemble-based algorithms composed of multiple classifiers have been developed. One such example is the Random Forest (RF) algorithm. Each decision tree in the forest produces a classification outcome, and the most frequent class prediction across all trees is selected as the final result (Figure 1). RF demonstrates high classification accuracy, is robust to outliers and noise, and is inherently resistant to overfitting [14].

The classification and regression trees used in Random Forest (RF) operate by performing data-driven classification. These trees are constructed by processing historical data to generate decision trees, which are subsequently used to categorise new data instances.

**Figure 2:**
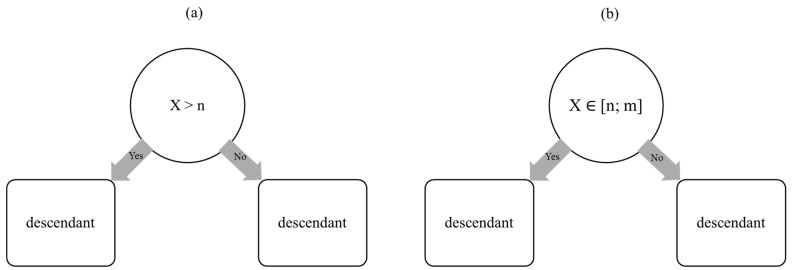
Principle of work of decision trees in (**a**) Random Forest Classifier and (**b**) Random Forest Regressor (source: authors).

A distinctive feature of decision trees is the use of queries that recursively partition the training data into increasingly smaller subsets. The set of queries consists exclusively of binary (“yes” or “no”) questions (Figure 2) [15].

By combining multiple decision trees, Random Forest (RF) reduces correlation among data samples by randomly selecting both instances and features. Initially, an equal number of samples and features are chosen randomly—the samples are used for training, while the features serve to construct the decision trees. This strategy reduces the likelihood of overfitting errors by minimising data redundancy, thereby increasing the model’s accuracy [16]. The partitioning of data enhances overall model performance by improving the algorithm’s efficiency in data processing and RF-based prediction. Properly processed (and partitioned) data simplify analytical tasks by supporting the identification of patterns and trends. Additionally, RF allows for parallelisation, which increases computational speed.

RF can be applied across various domains: banking—to assess the creditworthiness of loan applicants; healthcare—to assist in patient diagnosis through medical history analysis, and to optimise dosing regimens based on prior treatments; stock markets—to select appropriate markets or predict stock behaviour; and e-commerce—to forecast customer behaviour and preferences based on past purchases [17].

Random Forest Classifiers are particularly well-suited for detecting violations of established drug-likeness rules (Ro5, Muegge, bRo5) due to their capacity to model complex, nonlinear relationships among multiple molecular descriptors, high resilience to noisy and high-dimensional datasets, and demonstrated performance in ADME virtual screening contexts. Ensemble methods such as RF have shown near-perfect classification accuracy and ROC-AUC scores (~99–99.9%) in published studies, outperforming single-tree or linear models [18]. Moreover, RF inherently provides feature importance metrics, allowing interpretability in terms of contributing molecular properties (e.g., LogP, molecular weight, H-bond counts). The flexibility in tuning ensemble size and depth makes RF both scalable and adaptable to diverse chemical datasets while maintaining robustness and computational efficiency [19,20].

Although violations of drug-likeness rules are defined via deterministic thresholds, the use of a Random Forest model allows for a data-driven approach that captures nonlinear relationships, accommodates noisy or ambiguous inputs, and offers probabilistic outputs. Unlike rule-based scripts that rely solely on predefined cut-offs, machine learning models such as Random Forests can generalise beyond hard thresholds, enabling flexible prediction and better adaptation to real-world molecular variability.

The relevance of machine learning models lies in their ability to go beyond fixed rule-based systems by learning patterns from data, handling noisy or borderline compounds, and providing probabilistic outputs. Such models can generalise molecular behaviour more effectively, making them particularly useful in drug-likeness assessment where strict thresholds may not capture all relevant chemical variability [21,22,23].

The current study aims to evaluate newly created for the first time ML models, which use a Random Forest algorithm to predict violations of the rules for drug-likeness of peptides and their possible oral administration. Depending on the performance of the models they can be included in ML workflow for evaluating the drug-likeness of newly synthesised peptide molecules. Also, we aim to create and evaluate a model which gives information about violations of bRo5—strongly related with peptide molecules.

## 2. Results

### 2.1. Metrics of the Created Models

The evaluation of performance metrics for each model yielded the results presented in Table 1.

The obtained data indicate that all newly developed models exhibit high efficiency and accuracy, demonstrating their suitability for this study.

**Figure 3:**
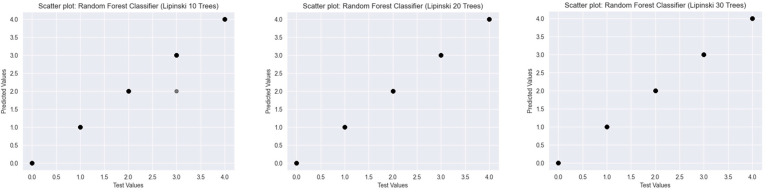
Scatter plots of Random Forest Classifiers descriptor-based models evaluating Ro5 (source: authors).

**Figure 4:**
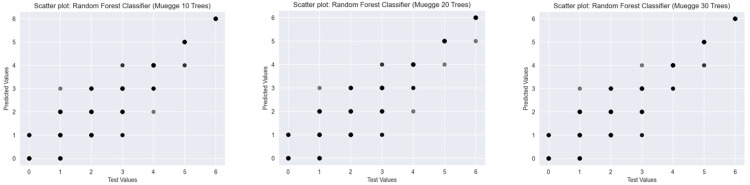
Scatter plots of Random Forest Classifiers descriptor-based models evaluating Muegge’s rule (source: authors).

**Figure 5:**
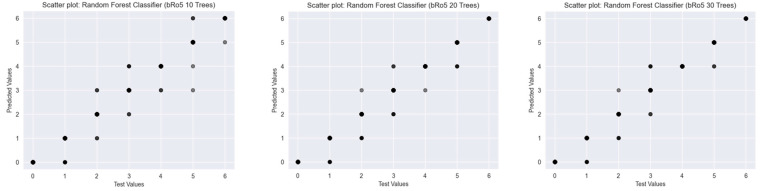
Scatter plots of Random Forest Classifiers descriptor-based models evaluating bRo5 (source: authors).

To assess the predictive performance of Random Forest Classifiers with varying numbers of decision trees (10, 20, and 30), scatter plots were generated for three established drug-likeness rules: Ro5 (Figure 3), the Muegge rule (Figure 4), and bRo5 (Figure 5). Each scatter plot displays the relationship between the true class values (*x*-axis) and the model’s predicted outputs (*y*-axis).

For the Ro5, increasing the number of trees from 10 to 20 significantly improved predictive alignment, with all predicted values in the 20- and 30-tree models aligning precisely along the diagonal line, indicating perfect classification. The 10-tree model exhibited minor deviations, suggesting underfitting at lower model complexity.

In the case of the Muegge rule, a higher level of class variability was present, reflected in the broader range of predicted values. The 10-tree model displayed notable dispersion from the ideal diagonal, while the 20- and 30-tree models demonstrated progressively better alignment. Nonetheless, even with 30 trees, minor deviations were observed, indicating that model performance may benefit from further optimisation or additional training data.

For the bRo5, a similar trend was observed. The 10-tree model showed considerable scatter, suggesting insufficient model capacity. The 20-tree model demonstrated improved predictive consistency, and the 30-tree variant further enhanced this performance, with predictions closely approximating the ideal diagonal line. However, small errors remained, particularly in mid-range class values, hinting at challenges in boundary classification.

Overall, the results indicate that increasing the number of trees in the Random Forest classifier improves model accuracy and generalisation, especially in rules with higher class diversity. While 20 trees appear sufficient for the Ro5, the more complex Muegge and bRo5 rules benefit from higher model capacity.

In addition to classification-based analysis, Random Forest Regressor models were trained and evaluated using three widely accepted drug-likeness rules: Ro5, Muegge, and bRo5. For each rule, models with 10, 20, and 30 trees were developed. Scatter plots depicting the relationship between true and predicted values were analysed to assess model performance and generalisation.

**Figure 6:**
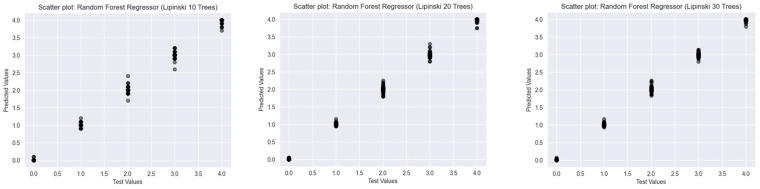
Scatter plots of Random Forest Regressors descriptor-based models evaluating Ro5. (source: authors).

The Ro5 (Figure 6), which featured a narrower target range (0–4), presented more visible variance across all models. The 10-tree regressor showed a wider spread around each class value, whereas the 20- and 30-tree models demonstrated tighter clustering. Nevertheless, slight underfitting around lower predicted values was present, suggesting that additional feature tuning or alternative model types may be needed for further refinement.

**Figure 7:**
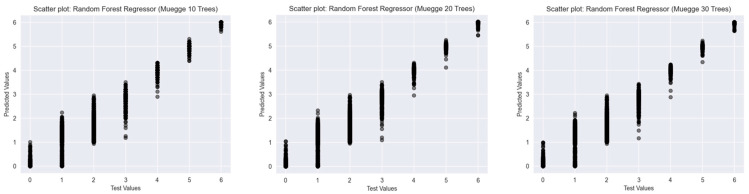
Scatter plots of Random Forest Regressors descriptor-based models evaluating Muegge’s rule (source: authors).

For the Muegge rule (Figure 7), all three models demonstrated a strong linear trend, with predicted values clustering tightly along the ideal diagonal line, particularly at extreme ends of the target range. Increasing the number of trees from 10 to 30 reduced the dispersion of predictions around mid-range values, indicating improved consistency. The 30-tree model achieved the most balanced distribution, suggesting enhanced regression stability.

**Figure 8:**
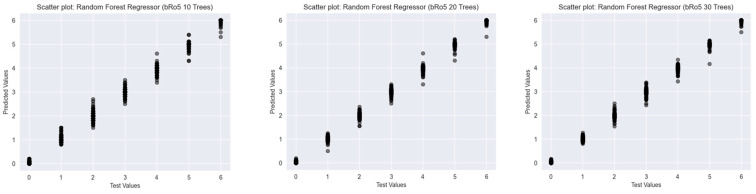
Scatter plots of Random Forest Regressors descriptor-based models evaluating bRo5 (source: authors).

In the case of the bRo5 (Figure 8), similar improvements were observed as the number of trees increased. The 10-tree model exhibited mild deviations and clustering inconsistencies, particularly in the mid to upper value ranges. These discrepancies diminished in the 20- and 30-tree models, with the latter providing smoother, more concentrated predictions around the true values, indicating improved performance.

Overall, the use of a greater number of trees in the Random Forest Regressor led to improved predictive precision, reduced variance, and stronger alignment with actual values across all three drug-likeness rules. These findings highlight the robustness of ensemble-based regression approaches in modelling graded rule adherence in cheminformatics tasks.

**Figure 9:**
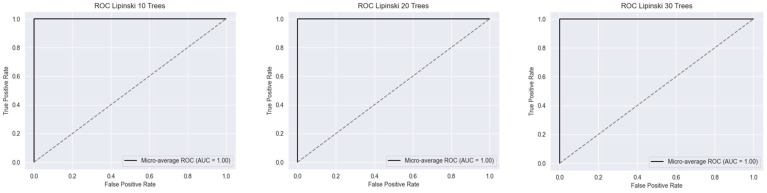
ROC curves of Random Forest Classifiers descriptor-based models evaluating Ro5 (source: authors).

**Figure 10:**
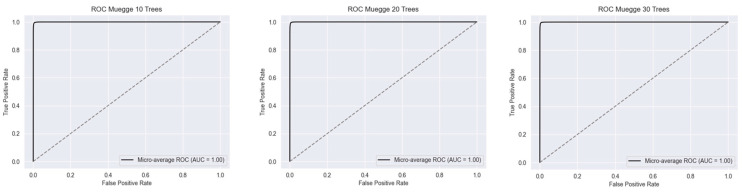
ROC curves of Random Forest Classifiers descriptor-based models evaluating Muegge’s rule (source: authors).

**Figure 11:**
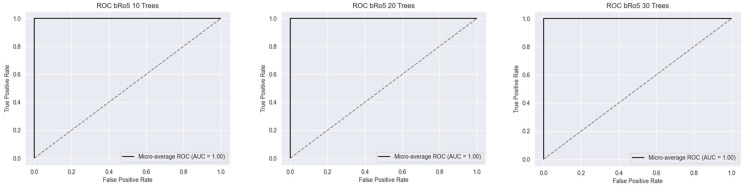
ROC curves of Random Forest Classifiers descriptor-based models evaluating bRo5 (source: authors).

To further validate the classification performance of the Random Forest models, Receiver Operating Characteristic (ROC) curves were generated for each drug-likeness rule (Ro5 (Figure 9), Muegge (Figure 10), and bRo5 (Figure 11)) using classifiers with 10, 20, and 30 trees. The ROC curve provides a graphical representation of the trade-off between true positive rate (sensitivity) and false positive rate across different decision thresholds. The area under the curve (AUC) serves as a quantitative measure of a model’s discriminative power, with values closer to 1.0 indicating superior classification performance.

All nine models, regardless of the number of trees or the rule applied, achieved a micro-average AUC of 1.00, signifying perfect separation between the positive and negative classes on the test data. The ROC curves in each case ascend rapidly to the top-left corner, a characteristic of high-performing classifiers with minimal false positives and false negatives.

The consistency of performance across all models and rules indicates a robust fit of the classifiers to the data, with no evident gain from increasing the number of trees beyond 10 under the current data conditions. This suggests that the feature space is highly separable for the tested rules, allowing even lower-complexity Random Forest models to achieve optimal classification.

Overall, the ROC curve analysis demonstrates that all Random Forest Classifiers exhibit excellent diagnostic accuracy, and increasing the number of trees from 10 to 30 does not substantially alter the classification outcome when evaluated via AUC.

### 2.2. Results of the Predictions

This study analyses the performance of Random Forest models—both classification and regression—in predicting the number of violations of three widely used rules for evaluating drug-likeness in peptide molecules: Ro5, bRo5, and Muegge’s rule. To assess the accuracy of the predictions, the results were compared with established references, including web-based platforms such as SwissADME and Molinspiration, as well as manually calculated values.

#### 2.2.1. Lipinski’s Rule of Five

Table 2 and Table 3 present the prediction results for Ro5 violations, as generated by the newly developed models, and compare them with those obtained from publicly available platforms—SwissADME and Molinspiration.

The conducted analysis shows that both models demonstrate exceptionally high consistency and stability in predicting the number of violations of Lipinski’s Rule of Five.

Regardless of the number of decision trees used (10, 20, or 30), the predictions for 23 out of 26 peptide molecules matched exactly in terms of violation counts with both online platforms used for comparison. For molecules **ML18 to ML22** and **ML24 to ML26**, the ML models predicted one additional violation compared to the reference platforms. This discrepancy may stem from stricter internal thresholds embedded in the training data, as well as the increased structural complexity of these molecules. It is important to note that for molecules **ML24 to ML26**, no comparison with SwissADME values was possible, as the platform is unable to process molecules with longer SMILES strings—i.e., larger molecular structures.

These findings suggest that both the classifier and regressor, even when using a small number of decision trees, are capable of accurately predicting violations of Lipinski’s rule.

#### 2.2.2. Muegge’s Rule

Table 4 and Table 5 present the prediction results from the RF Classifier (Table 4) and RF Regressor (Table 5) models regarding violations of Muegge’s rule. In these tables, the results are compared exclusively with the web-based platform SwissADME, as Molinspiration provides calculations only for Ro5 violations.

The predictions of Muegge’s rule violations using the Random Forest models exhibit consistency across different numbers of estimators and strong agreement between the classifier and regressor. The comparison between model predictions and SwissADME outputs indicates that while the Random Forest models tend to slightly underestimate the number of Muegge’s rule violations (by approximately one violation in several cases), they exhibit strong internal consistency and predictive alignment across different estimators.

#### 2.2.3. Beyond Lipinski’s Rule of Five

Table 6 presents the RF Classifier model, and Table 7 presents the RF Regressor model, both designed to predict violations of the bRo5, which applies to peptide molecules. It is essential to emphasise that these six newly developed RF models represent the first in silico tools used to predict bRo5 values, making their comparative evaluation a crucial component of this study. Given the novelty of these models, their predictions were compared against manually verified values of the molecular descriptors for the peptide molecules under investigation.

The prediction results demonstrate excellent performance, with both types of RF algorithms producing accurate outcomes. The classifier and regressor showed near-complete agreement with the manually calculated violation counts, indicating that the models effectively capture deviation thresholds and possess the capability to make fine-grained predictions.

The only exception occurred with molecule 20, where the regressor consistently predicted three violations instead of four, regardless of the number of decision trees. Additionally, for molecule **ML24**, the RF Regressor with a higher number of trees failed to predict the correct number of violations. On the classifier side, incorrect predictions were observed for molecule **ML20** with 10 trees, and peptide molecules **ML21** and **ML22** with 20 trees.

#### 2.2.4. Classifiers Working with Fingerprint Representations

To evaluate the impact of input data (training dataset and tested peptide molecules) on the accuracy of rule violation prediction, we compared Random Forest Classifiers trained on classical molecular descriptors to those trained on vectorized SMILES representations using Morgan fingerprints.

The performance metrics of the Random Forest classification models (Table 8) across the three rule-based filters (Ro5, bRo5, and Muegge) demonstrate consistently high predictive accuracy. Models trained to predict Ro5 and bRo5 violations achieved near-perfect performance across all metrics, with accuracy, precision, recall, and F1-score values exceeding 0.98, regardless of the number of estimators used. In contrast, predictions for the Muegge rule showed slightly lower but still strong performance, with all metrics ranging from 0.89 to 0.902. Notably, increasing the number of estimators from 10 to 30 yielded marginal improvements across all rules, indicating that model performance is stable and robust even with a moderate number of trees.

The models based on fingerprint representations exhibited poorer alignment with the reference rule violations (Table 9, Table 10 and Table 11). In particular, a small number of the fingerprint-based predictions matched the reference outputs, regardless of the number of estimators used. In contrast, models trained on physicochemical descriptors produced several exact matches, especially for the Ro5 and bRo5 rules, which are defined by explicit threshold-based criteria.

These findings suggest that, while structural fingerprints may encode substructural features, they are less effective than direct molecular descriptors when the task involves predicting rule violations defined by deterministic and threshold-driven logic. The descriptor-based models appear to be more interpretable and better suited for tasks where the prediction target is essentially a recoding of the molecular properties of peptide-based compounds.

Poor results of the classifier models led us to the decision not to try to evaluate the performance of regression models working with fingerprints, to predict violations of three rules for drug-likeness.

## 3. Discussion

The results obtained in this study highlight the robustness and applicability of RF models in the context of rule-based drug-likeness prediction, specifically for peptide-based molecules. The evaluation metrics of all developed models indicate excellent performance, with most classification and regression scores approaching or reaching optimal values (i.e., close to 1). This not only reflects the effectiveness of the model training pipeline but also demonstrates the appropriateness of the curated dataset.

A comparative analysis between classifier and regressor configurations across three major rule frameworks—Ro5, bRo5, and Muegge’s rule—revealed a consistently high level of agreement. The models remained stable across different numbers of decision trees (*n* = 10, 20, 30), indicating that the task of predicting rule violations is well-defined and generalizable even in computationally simplified settings. This further supports the interpretability and scalability of RF architectures in cheminformatics applications.

In the context of Ro5, the RF classifier and regressor models demonstrated remarkable predictive consistency and robustness. Across the majority of cases, 23 out of 26 peptide molecules, the predicted number of rule violations matched exactly with outputs from two widely used reference platforms, SwissADME and Molinspiration. This agreement remained stable across varying model complexities, regardless of the number of decision trees employed (10, 20, or 30). For a limited subset of molecules (**ML18** to **ML22** and **ML24** to **ML26**), the models predicted one additional violation relative to the benchmarks. These discrepancies may arise from more stringent internal thresholds derived from the training dataset or the greater molecular complexity and size of the peptides in question. Notably, SwissADME was unable to process molecules **ML24** to **ML26** due to limitations in handling extended SMILES strings, which precluded a direct comparison for these entries. Overall, the findings affirm that both RF models, even in relatively simple configurations, are well-suited for accurately identifying Ro5 violations and thus offer a reliable framework for early-stage drug-likeness assessment.

The performance of the models with respect to Muegge’s rule, which is more descriptor-intensive than Ro5, warrants special attention. While the RF models exhibited strong internal consistency and minimal variation among configurations, a systematic underestimation of approximately one violation was observed when compared with SwissADME outputs. This underestimation can be primarily attributed to two sources: (1) differences in descriptor calculation methodologies between RDKit (used in this study) and the SwissADME backend, particularly for logP and TPSA; (2) the absence of specific key descriptors—most notably, heteroatom count—from the training dataset. These factors likely contributed to boundary-level misclassification in some peptide structures. Despite these limitations, the models retained high predictive reliability, correctly ranking peptides based on their relative risk of rule violation. This suggests that the RF approach captures the broader trends in physicochemical profile distribution, even when exact matches are not achieved. Future work should incorporate heteroatom-related features and harmonise descriptor calculations across platforms to minimise systematic bias and enhance model granularity.

In the case of the bRo5, designed to accommodate the inherent complexity of peptide-based compounds, the models achieved near-perfect alignment with manually calculated reference values. This affirms the suitability of bRo5 as a rule framework for peptide evaluation and highlights the adaptability of ML systems to non-traditional chemical scaffolds. An interesting observation is the minimal divergence in performance between classifiers and regressors, despite their distinct computational logic. Given the limited range of possible rule violations (typically 0–6), both types of models converged toward similar predictions. This suggests that for discrete, low-dimensional classification tasks in cheminformatics, model type selection may be guided more by interpretability or integration needs than by performance differentials.

From the perspective of peptide pharmacology, the oral bioavailability of peptide-based drugs remains a critical barrier in therapeutic development. Traditional rule-based heuristics, such as Ro5, are generally too restrictive to encompass the structural and physicochemical diversity of bioactive peptides. This study demonstrates that when extended or adapted rules, such as bRo5, are employed, peptide drug-likeness becomes more tractable using ML-based predictive frameworks. The ability of the RF models to reliably predict bRo5 compliance in larger peptide structures, often regarded as challenging for oral administration, underscores their utility in early-stage candidate filtering. As such, these models may serve as a valuable tool in narrowing down peptide libraries to identify molecules with improved absorption potential.

Importantly, four of the studied peptide molecules (**ML13** to **ML16**) fully complied with both Ro5 and bRo5 frameworks, with only a single discrepancy noted in **ML15**. These molecules represent promising candidates for oral formulation development, as their descriptor profiles are within the bioavailability-friendly boundaries defined by both classical and peptide-adapted rules. Follow-up research on their pharmacokinetics and toxicity profiles is warranted.

To address the conceptual concern regarding the determinism of the prediction task, we conducted an additional comparative analysis using alternative molecular representations. In particular, we trained Random Forest Classifiers using Morgan fingerprints instead of physicochemical descriptors, and evaluated their ability to predict rule violations for Ro5, bRo5, and Muegge criteria on a test set of peptide molecules. This comparison is especially relevant in the context of peptide-based compounds, where the bRo5 rule is of higher importance due to the common exceedance of Ro5 thresholds in peptides.

The fingerprint-based models, however, failed to produce a significant number of exact matches with the reference rule violations, regardless of the number of estimators used. In contrast, descriptor-based models yielded multiple correct predictions, particularly for bRo5 and Ro5 filters. The choice of input representation critically affects the model’s capacity to capture the underlying rule logic. They also refute the notion that the ML model is merely re-encoding deterministic thresholds, as performance is clearly impacted by the format and semantic content of the input features. Thus, this empirical evaluation affirms the relevance of ML models, particularly for tasks involving flexible or multi-criteria decision rules as observed in the bRo5 domain of peptide therapeutics.

Deterministic rule application assumes descriptor calculation is always consistent. However, in cheminformatics workflows, SMILES strings can lead to different descriptor values depending on software or preprocessing. An ML model trained on the output of such workflows can “learn” to be robust to such variance and be more generalizable than a brittle rule-based script.

The RF model, even when trained on rule-defined descriptors, can identify complex patterns, interactions, or correlations that are not captured by simple rule-based logic, even in noisy, incomplete, or ambiguous real-world molecular datasets. For instance, the model may prioritise specific descriptor combinations, account for redundancy, or implicitly weight the importance of certain features in a way that reveals hidden structure in the dataset.

Overall, this study demonstrates the potential of Random Forest models as automated in silico filters for drug-likeness evaluation, particularly in peptide-focused pipelines where conventional heuristics often fail. These results support further integration of AI-driven methodologies into the early stages of drug discovery and molecular prioritisation.

Future directions include the expansion of the training dataset to encompass molecules with intermediate rule violation counts (e.g., 1–2), incorporation of additional structural descriptors, and benchmarking RF models against other state-of-the-art ML algorithms such as XGBoost and Gradient Boosting Machines (GBMs). Such developments may further enhance prediction accuracy, sensitivity, and the broader applicability of AI in rational drug design.

## 4. Materials and Methods

For the purpose of our study we used SMILES strings of 26 peptide molecules (ML1–ML26), which were previously synthesised by the authors and contain non-proteinogenic amino acids, and conjugates of some of them, are investigated.

### 4.1. Software and Databases

The present in silico investigation utilised the PubChem^®^ database (https://pubchem.ncbi.nlm.nih.gov, accessed on 4 June 2025) [24] to collect data, RDKit library (https://www.rdkit.org, accessed on 5 June 2025) [25] to access molecular descriptors of tested molecules and web-based software platforms, SwissADME (http://www.swissadme.ch, accessed on 8 July 2025) [26] and Molinspiration (https://www.molinspiration.com, accessed on 18 November 2021) [27] to obtain control data for comparison.

### 4.2. Study Design

(1) The models were trained using a dataset obtained from the PubChem database, which contains information on the chemical and physicochemical properties of drug and non-drug molecules.
(2) The PubChem dataset (containing >300,000 drug (small and peptide) and non-drug molecules) was preprocessed to meet the objectives of the study. Only the following data were retained: molecule name, SMILES string, and values of molecular descriptors included in Lipinski’s rule (molecular weight, logP, number of hydrogen bond donors and acceptors), along with TPSA and rotatable bonds.
(3) Three different counters were developed to quantify violations of Ro5, bRo5, and the criteria proposed by Muegge et al. [12] for the included molecules. The resulting values were added to a new dataset, which was subsequently used to train the ML models.
(4) As the Random Forest Classifier is a supervised learning algorithm, the newly generated dataset was split into features (training data) and target values. The molecular descriptor values were used as input features, while the number of rule violations—calculated using the custom counters—served as target outputs. This dataset was further subdivided into training and evaluation sets to measure model performance, by train_test_split function which is included in scikit-learn. The parameters of splitting the data were test_size = 0.2 and random_state = 42. Data leakage prevented by applying strict train-validation-test separation prior to feature engineering, ensuring that all preprocessing steps were fitted only on the training data and applied to test data in a pipeline setting.
(5) Following the data split, models were trained separately for each of the evaluated rules. Specifically, for Ro5, bRo5, and Muegge’s criteria, three models were created per rule using 10, 20, and 30 decision trees, respectively. We used grid search to help in optimising of hyper parameters values which was used. Especially tested hyper parameters was max_features = ‘sqrt’ or ‘log2’, min_samples_leaf = 1, min_samples_split = 2 or 5, but results yielded by default values of hyper parameters was better.
(6) After training, model performance was evaluated using standard metrics to identify and potentially discard underperforming models.
(7) To predict the number of rule violations for the studied peptide molecules, their molecular descriptor values first had to be determined. For this purpose, the RDKit library (https://www.rdkit.org, accessed on 5 June 2025) for Python 3.13 was used to calculate molecular descriptors from their SMILES strings. The resulting data were compiled into a test dataset containing descriptor values for all peptide candidates.
(8) Once the models were trained and the test dataset constructed, predictions were made regarding violations of Ro5, bRo5, and Muegge’s rule for drug-likeness. The predicted values were then aggregated and compared with rule violation data obtained from two web-based platforms: SwissADME and Molinspiration. The SwissADME values were generated after model training, while the Molinspiration data were obtained in 2020.
(9) For creating a models for comparison, we extract information about Morgan fingerprints from SMILES strings included in the training dataset. We combine them with the already calculated number of violations of each rule. After that we used again train_test_split function again, like in step 4.
(10) Differentiated data were used for training of models (Random Forest Classifiers) with different numbers of estimators and evaluating their performance before predictions.
(11) The next step was to obtain fingerprint representation of tested molecules and incorporating them in new dataset.
(12) The final stage was the prediction of violations and comparing them with web-based platforms or manually calculated.

### 4.3. Evaluation of Created Classifier Models

Depending on the specific task addressed by the supervised ML model, appropriate evaluation metrics can be selected [28].

In classification tasks, the goal is to predict whether an instance belongs to a positive or negative class. As such, each prediction falls into one of four categories: True Positive (TP): a correctly predicted positive outcome; True Negative (TN): a correctly predicted negative outcome; False Positive (FP): a negative instance incorrectly classified as positive; False Negative (FN): a positive instance incorrectly classified as negative [29].

Evaluating the performance of classification models is critical for determining their effectiveness across various applications. The key metrics used to assess classification models, along with their respective formulas, are as follows:

Accuracy represents the proportion of correctly classified instances and reflects the overall effectiveness of the model:

$$Accuracy=\frac{TP+TN}{TP+TN+FP+FN}\in \left[0,1\right]$$

Precision quantifies the model’s ability to identify positive instances while minimising false positives correctly:

$$Precision=\frac{TP}{TP+FP}\in \left[0,1\right]$$

Sensitivity (Recall) measures the model’s ability to detect positive outcomes, thereby reducing false negatives:

$$Recall=\frac{TP}{TP+FN}\in \left[0,1\right]$$

F1-score evaluates the model’s performance by harmonising both precision and recall [30]:

$$F1-score=\frac{2\times Precision\times Recall}{Precision+Recall}\in \left[0,1\right]$$

For each individual cut-off point, there is a corresponding pair of diagnostic sensitivity and specificity values. To construct a ROC (Receiver Operating Characteristic) curve, these pairs are plotted with 1 − specificity on the *x*-axis and sensitivity on the *y*-axis. The shape of the ROC curve and the area under the curve (AUC) provide valuable insights into the test’s ability to distinguish between different classes included in the study. The closer the curve is to the upper-left corner of the graph, and the larger the AUC, the better the test performs in terms of discrimination. The AUC ranges from 0 to 1, where AUC = 1.0 indicates a perfect diagnostic test, AUC = 0.5 suggests no discriminative power—equivalent to random guessing. Thus, the AUC serves as a reliable indicator of the overall diagnostic accuracy of the test.

### 4.4. Evaluation of Created Regressor Models

Regression algorithms represent a class of ML approaches that model the relationship between a dependent variable and one or more independent variables. These algorithms are widely used in data analysis and predictive modelling. The primary metrics for evaluating the performance of regression models include:

Mean Squared Error (MSE) measures the precision of the model by calculating the average of the squared differences between predicted and actual values:

$$MSE=\frac{1}{n}\sum_{i=1}^{n}{\left(x_{i}-y_{i}\right)}^{2}\in [0,\infty )$$

Mean Absolute Error (MAE) quantifies the average absolute difference between the predicted and actual values:

$$MAE=\frac{1}{n}\sum_{i=1}^{n}\left|x_{i}-y_{i}\right|\in [0,\infty )$$

R-squared coefficient (R^2^) indicates the proportion of the variance in the dependent variable that the independent variables can explain [30]:

$$R^{2}=1-\frac{MSE}{Var\left(y\right)}\in [0,1]$$

## 5. Conclusions

In conclusion, Random Forest models show exceptional promise for predicting drug-likeness rule violations in peptide-based compounds, offering strong accuracy, stability, and generalizability. Their capacity to process extended molecular rules, such as bRo5, and to align with expert-calculated and SwissADME results positions them as valuable tools in AI-assisted peptide drug discovery. The models’ applicability to structurally complex peptides also holds promise for advancing oral peptide therapeutics, supporting their future inclusion in early-stage screening platforms.

While this study focuses on well-established drug-likeness rules, our methodology opens the door to applying ML to more subjective or experimentally derived thresholds, for example, ADME of peptide-based compounds, where cut-offs are not universally defined. We have positioned this work as a first step toward such applications.

## Figures and Tables

**Table 1:** Evaluated metrics of created Random Forest descriptor-based models.

	RF Model (Number of Estimators)								
Metrics	Ro5 (10)	Ro5 (20)	Ro5 (30)	bRo5 (10)	bRo5 (20)	bRo5 (30)	Muegge (10)	Muegge (20)	Muegge (30)
**Classification models**									
Accuracy	1.0	1.0	1.0	0.999	0.999	0.999	0.985	0.986	0.986
Precision	1.0	1.0	1.0	0.999	0.999	0.999	0.985	0.986	0.986
Sensitivity (Recall)	1.0	1.0	1.0	0.999	0.999	0.999	0.985	0.986	0.986
F1-score	1.0	1.0	1.0	0.999	0.999	0.999	0.985	0.986	0.986
**Regression models**									
MSE	0.0	0.0	5.46 × 10^−8^	7.30 × 10^−5^	8.85 × 10^−5^	6.21 × 10^−5^	0.01	0.01	0.01
MAE	0.0	0.0	1.63 × 10^−6^	0.0002	0.0002	0.0002	0.019	0.019	0.019
R^2^	1.0	1.0	0.999	0.999	0.999	0.999	0.996	0.996	0.996

**Table 2:** Predictions of the number of Ro5 violations from the Random Forest Classifier descriptor-based model.

Peptide Molecule	Violations Ro5 (Estimators = 10)	Violations Ro5 (Estimators = 20)	Violations Ro5 (Estimators = 30)	Violations Ro5 (SwissADME)	Violations Ro5 (Molinspiration)
ML 1	2	2	2	2	2
ML 2	2	2	2	2	2
ML 3	2	2	2	2	2
ML 4	2	2	2	2	2
ML 5	2	2	2	2	2
ML 6	2	2	2	2	2
ML 7	2	2	2	2	2
ML 8	2	2	2	2	2
ML 9	2	2	2	2	2
ML 10	2	2	2	2	2
ML 11	2	2	2	2	2
ML 12	2	2	2	2	2
ML 13	0	0	0	0	0
ML 14	0	0	0	0	0
ML 15	1	1	1	1	1
ML 16	0	0	0	0	0
ML 17	3	3	3	3	3
ML 18	4	4	4	3	3
ML 19	4	4	4	3	3
ML 20	4	4	4	3	3
ML 21	4	4	4	3	3
ML 22	4	4	4	3	3
ML 23	3	3	3	3	3
ML 24	4	4	4	*NaN*	3
ML 25	4	4	4	*NaN*	3
ML 26	4	4	4	*NaN*	3

*Note: Grey is an indicator for exact matches*.

**Table 3:** Predictions of the number of Ro5 violations from the Random Forest Regressor descriptor-based model.

Peptide Molecule	Violations Ro5 (Estimators = 10)	Violations Ro5 (Estimators = 20)	Violations Ro5 (Estimators = 30)	Violations Ro5 (SwissADME)	Violations Ro5 (Molinspiration)
ML 1	2	2	2	2	2
ML 2	2	2	2	2	2
ML 3	2	2	2	2	2
ML 4	2	2	2	2	2
ML 5	2	2	2	2	2
ML 6	2	2	2	2	2
ML 7	2	2	2	2	2
ML 8	2	2	2	2	2
ML 9	2	2	2	2	2
ML 10	2	2	2	2	2
ML 11	2	2	2	2	2
ML 12	2	2	2	2	2
ML 13	0	0	0	0	0
ML 14	0	0	0	0	0
ML 15	1	1	1	1	1
ML 16	0	0	0	0	0
ML 17	3	3	3	3	3
ML 18	4	4	4	3	3
ML 19	4	4	4	3	3
ML 20	4	4	4	3	3
ML 21	4	4	4	3	3
ML 22	4	4	4	3	3
ML 23	3	3	3	3	3
ML 24	4	4	4	*NaN*	3
ML 25	4	4	4	*NaN*	3
ML 26	4	4	4	*NaN*	3

*Note: Grey is an indicator for exact matches*.

**Table 4:** Predictions of the number of Muegge’s rule violations from the Random Forest Classifier descriptor-based model.

Peptide Molecule	Violations of Muegge’s Rule (Estimators = 10)	Violations of Muegge’s Rule (Estimators = 20)	Violations of Muegge’s Rule (Estimators = 30)	Violations of Muegge’s Rule (SwissADME)
ML 1	3	3	3	3
ML 2	3	3	3	3
ML 3	3	3	3	3
ML 4	3	3	3	3
ML 5	3	3	3	4
ML 6	3	3	3	4
ML 7	3	3	3	4
ML 8	3	3	3	4
ML 9	3	3	3	3
ML 10	3	3	3	4
ML 11	3	3	3	4
ML 12	3	3	3	4
ML 13	0	0	0	1
ML 14	0	0	0	0
ML 15	0	0	0	0
ML 16	0	0	0	1
ML 17	4	4	4	6
ML 18	5	5	5	5
ML 19	5	5	5	5
ML 20	6	6	6	6
ML 21	5	5	5	6
ML 22	5	5	5	6
ML 23	4	4	4	5

*Note: Grey is an indicator for exact matches*.

**Table 5:** Predictions of the number of Muegge’s rule violations from the Random Forest Regressor descriptor-based model.

Peptide Molecule	Violations of Muegge’s Rule (Estimators = 10)	Violations of Muegge’s Rule (Estimators = 20)	Violations of Muegge’s Rule (Estimators = 30)	Violations of Muegge’s Rule (SwissADME)
ML 1	3	3	3	3
ML 2	3	3	3	3
ML 3	3	3	3	3
ML 4	3	3	3	3
ML 5	3	3	3	4
ML 6	3	3	3	4
ML 7	3	3	3	4
ML 8	3	3	3	4
ML 9	3	3	3	3
ML 10	3	3	3	4
ML 11	3	3	3	4
ML 12	3	3	3	4
ML 13	0	0	0	1
ML 14	0	0	0	0
ML 15	0	0	0	0
ML 16	0	0	0	1
ML 17	4	4	4	6
ML 18	5	5	5	5
ML 19	5	5	5	5
ML 20	5	5	5	6
ML 21	5	5	5	6
ML 22	5	5	5	6
ML 23	4	4	4	5

*Note: Grey is an indicator for exact matches*.

**Table 6:** Predictions of the number of bRo5 violations from the Random Forest Classifier descriptor-based model.

**Peptide** **Molecule**	**Violations of** **bRo5** **(Estimators = 10)**	**Violations of** **bRo5** **(Estimators = 20)**	**Violations of** **bRo5** **(Estimators = 30)**	**Manual**
ML 1	2	2	2	2
ML 2	2	2	2	2
ML 3	2	2	2	2
ML 4	2	2	2	2
ML 5	2	2	2	2
ML 6	2	2	2	2
ML 7	2	2	2	2
ML 8	2	2	2	2
ML 9	2	2	2	2
ML 10	2	2	2	2
ML 11	2	2	2	2
ML 12	2	2	2	2
ML 13	0	0	0	0
ML 14	0	0	0	0
ML 15	0	0	0	0
ML 16	0	0	0	0
ML 17	3	3	3	3
ML 18	3	3	3	3
ML 19	3	3	3	3
ML 20	3	4	4	4
ML 21	3	4	3	3
ML 22	3	4	3	3
ML 23	3	3	3	3
ML 24	6	6	6	6
ML 25	5	5	5	5
ML 26	4	4	4	4

*Note: Grey is an indicator for exact matches*.

**Table 7:** Predictions of the number of bRo5 violations from the Random Forest Regressor descriptor-based model.

Peptide Molecule	Violations of bRo5 (Estimators = 10)	Violations of bRo5 (Estimators = 20)	Violations of bRo5 (Estimators = 30)	Manual
ML 1	2	2	2	2
ML 2	2	2	2	2
ML 3	2	2	2	2
ML 4	2	2	2	2
ML 5	2	2	2	2
ML 6	2	2	2	2
ML 7	2	2	2	2
ML 8	2	2	2	2
ML 9	2	2	2	2
ML 10	2	2	2	2
ML 11	2	2	2	2
ML 12	2	2	2	2
ML 13	0	0	0	0
ML 14	0	0	0	0
ML 15	0	0	0	0
ML 16	0	0	0	0
ML 17	3	3	3	3
ML 18	3	3	3	3
ML 19	3	3	3	3
ML 20	3	3	3	4
ML 21	3	3	3	3
ML 22	3	3	3	3
ML 23	3	3	3	3
ML 24	6	5	5	6
ML 25	5	5	5	5
ML 26	4	4	4	4

*Note: Grey is an indicator for exact matches*.

**Table 8:** Evaluated metrics of created Random Forest models working with fingerprints.

	RF Model (Number of Estimators)								
Metrics	Ro5 (10)	Ro5 (20)	Ro5 (30)	bRo5 (10)	bRo5 (20)	bRo5 (30)	Muegge (10)	Muegge (20)	Muegge (30)
**Classification models**									
Accuracy	0.983	0.985	0.987	0.99	0.989	0.99	0.89	0.897	0.902
Precision	0.983	0.985	0.988	0.99	0.988	0.99	0.888	0.896	0.901
Sensitivity (Recall)	0.983	0.985	0.988	0.99	0.989	0.99	0.89	0.897	0.902
F1-score	0.983	0.985	0.988	0.99	0.988	0.990	0.889	0.896	0.901

**Table 9:** Predictions of the number of Ro5 violations from the Random Forest Classifier using Morgan fingerprints representation.

Peptide Molecule	Violations Ro5 (Estimators = 10)	Violations Ro5 (Estimators = 20)	Violations Ro5 (Estimators = 30)	Violations Ro5 (SwissADME)	Violations Ro5 (Molinspiration)
ML 1	0	0	0	2	2
ML 2	0	0	0	2	2
ML 3	0	0	0	2	2
ML 4	0	0	0	2	2
ML 5	0	0	0	2	2
ML 6	0	0	0	2	2
ML 7	0	0	0	2	2
ML 8	0	0	0	2	2
ML 9	0	0	0	2	2
ML 10	0	0	0	2	2
ML 11	0	0	0	2	2
ML 12	0	0	0	2	2
ML 13	0	0	0	0	0
ML 14	0	0	0	0	0
ML 15	0	0	0	1	1
ML 16	0	0	0	0	0
ML 17	0	0	0	3	3
ML 18	0	0	0	3	3
ML 19	0	0	0	3	3
ML 20	0	0	0	3	3
ML 21	0	0	0	3	3
ML 22	0	0	0	3	3
ML 23	0	0	0	3	3
ML 24	0	0	0	*NaN*	3
ML 25	0	0	0	*NaN*	3
ML 26	0	0	0	*NaN*	3

**Table 10:** Predictions of the number of Muegge’s rule violations from the Random Forest Classifier using Morgan fingerprints representation.

Peptide Molecule	Violations of Muegge’s Rule (Estimators = 10)	Violations of Muegge’s Rule (Estimators = 20)	Violations of Muegge’s Rule (Estimators = 30)	Violations of Muegge’s Rule (SwissADME)
ML 1	0	0	0	3
ML 2	0	1	0	3
ML 3	0	2	1	3
ML 4	1	1	0	3
ML 5	1	0	0	4
ML 6	1	0	0	4
ML 7	0	0	0	4
ML 8	1	0	0	4
ML 9	0	0	0	3
ML 10	0	0	0	4
ML 11	1	1	1	4
ML 12	1	1	1	4
ML 13	2	1	0	1
ML 14	0	1	0	0
ML 15	0	1	0	0
ML 16	0	1	0	1
ML 17	1	0	0	6
ML 18	0	0	0	5
ML 19	0	0	0	5
ML 20	0	0	0	6
ML 21	1	0	0	6
ML 22	1	0	0	6
ML 23	1	0	0	5

**Table 11:** Predictions of the number of bRo5 violations from the Random Forest Classifier using Morgan fingerprints representation.

Peptide Molecule	Violations of bRo5 (Estimators = 10)	Violations of bRo5 (Estimators = 20)	Violations of bRo5 (Estimators = 30)	Manual
ML 1	0	0	0	2
ML 2	0	0	0	2
ML 3	0	0	0	2
ML 4	0	0	0	2
ML 5	0	0	0	2
ML 6	0	0	0	2
ML 7	0	0	0	2
ML 8	0	0	0	2
ML 9	0	0	0	2
ML 10	0	0	0	2
ML 11	0	0	0	2
ML 12	0	0	0	2
ML 13	0	0	0	0
ML 14	0	0	0	0
ML 15	0	0	0	0
ML 16	0	0	0	0
ML 17	0	0	0	3
ML 18	0	0	0	3
ML 19	0	0	0	3
ML 20	0	0	0	4
ML 21	0	0	0	3
ML 22	0	0	0	3
ML 23	0	0	0	3
ML 24	0	0	0	6
ML 25	0	0	0	5
ML 26	0	0	0	4

## Data Availability

The original contributions presented in this study are included in the article. Further inquiries can be directed to the corresponding author. Further data at https://github.com/MLambev/drug_likeness_rules, accessed on 16 July 2025.

## References

[1] Lutz M.W., Menius J.A., Choi T.D., Gooding Laskody R., Domanico P.L., Goetz A.S., Saussy D.L. (1996). Experimental Design for High-Throughput Screening. Drug Discov. Today.

[2] Gallop M.A., Barrett R.W., Dower W.J., Fodor S.P.A., Gordon E.M. (1994). Applications of Combinatorial Technologies to Drug Discovery. 1. Background and Peptide Combinatorial Libraries. J. Med. Chem..

[3] Gordon E.M., Barrett R.W., Dower W.J., Fodor S.P.A., Gallop M.A. (1994). Applications of Combinatorial Technologies to Drug Discovery. 2. Combinatorial Organic Synthesis, Library Screening Strategies, and Future Directions. J. Med. Chem..

[4] Martin E.J., Blaney J.M., Siani M.A., Spellmeyer D.C., Wong A.K., Moos W.H. (1995). Measuring Diversity: Experimental Design of Combinatorial Libraries for Drug Discovery. J. Med. Chem..

[5] Warr W.A. (1997). Combinatorial Chemistry and Molecular Diversity. An Overview. J. Chem. Inf. Comput. Sci..

[6] Brown R.D., Martin Y.C. (1997). Designing Combinatorial Library Mixtures Using a Genetic Algorithm. J. Med. Chem..

[7] Pötter T., Matter H. (1998). Random or Rational Design? Evaluation of Diverse Compound Subsets from Chemical Structure Databases. J. Med. Chem..

[8] Clark D.E., Pickett S.D. (2000). Computational Methods for the Prediction of ‘Drug-Likeness’. Drug Discov. Today.

[9] Podlogar B.L., Muegge I., Brice L.J. (2001). Computational Methods to Estimate Drug Development Parameters. Curr. Opin. Drug Discov. Dev..

[10] Lipinski C.A., Lombardo F., Dominy B.W., Feeney P.J. (2001). Experimental and Computational Approaches to Estimate Solubility and Permeability in Drug Discovery and Development Settings. Adv. Drug Deliv. Rev..

[11] Doak B.C., Over B., Giordanetto F., Kihlberg J. (2014). Oral Druggable Space beyond the Rule of 5: Insights from Drugs and Clinical Candidates. Chem. Biol..

[12] Muegge I., Heald S.L., Brittelli D. (2001). Simple Selection Criteria for Drug-like Chemical Matter. J. Med. Chem..

[13] Bemis G.W., Murcko M.A. (1996). The Properties of Known Drugs. 1. Molecular Frameworks. J. Med. Chem..

[14] Liu Y., Wang Y., Zhang J., Liu B., Ma M., Chang J. (2012). New Machine Learning Algorithm: Random Forest. Information Computing and Applications.

[15] Timofeev R. (2004). Classification and Regression Trees (CART) Theory and Applications Theory and Applications. Master’s Thesis.

[16] Cutler A., Cutler D.R., Stevens J.R., Zhang C., Ma Y. (2012). Random Forests. Ensemble Machine Learning.

[17] Salman H.A., Kalakech A., Steiti A. (2024). Random Forest Algorithm Overview. Babylon. J. Mach. Learn..

[18] Nhlapho S., Nyathi M., Ngwenya B., Dube T., Telukdarie A., Munien I., Vermeulen A., Chude-Okonkwo U. (2024). Druggability of Pharmaceutical Compounds Using Lipinski Rules with Machine Learning. Sci. Pharm..

[19] Aqeel I., Majid A. (2022). Hybrid Approach to Identify Druglikeness Leading Compounds against COVID-19 3CL Protease. Pharmaceuticals.

[20] Mughal H., Wang H., Zimmerman M., Paradis M.D., Freundlich J.S. (2021). Random Forest Model Prediction of Compound Oral Exposure in the Mouse. ACS Pharmacol. Transl. Sci..

[21] Hu Q., Feng M., Lai L., Pei J. (2018). Prediction of Drug-Likeness Using Deep Autoencoder Neural Networks. Front. Genet..

[22] Abbas K., Abbasi A., Dong S., Niu L., Yu L., Chen B., Cai S.-M., Hasan Q. (2021). Application of Network Link Prediction in Drug Discovery. BMC Bioinform..

[23] Svetnik V., Liaw A., Tong C., Culberson J.C., Sheridan R.P., Feuston B.P. (2003). Random Forest: A Classification and Regression Tool for Compound Classification and QSAR Modeling. J. Chem. Inf. Comput. Sci..

[24] Kim S., Chen J., Cheng T., Gindulyte A., He J., He S., Li Q., Shoemaker B.A., Thiessen P.A., Yu B. (2025). PubChem 2025 Update. Nucleic Acids Res..

[25] Landrum G., Tosco P., Kelley B., Rodriguez R., Cosgrove D., Vianello R., Sriniker, Gedeck P., Jones G., Kawashima E. RDKit: Open-Source Cheminformatics Software. https://www.rdkit.org.

[26] Daina A., Michielin O., Zoete V. (2017). SwissADME: A Free Web Tool to Evaluate Pharmacokinetics, Drug-Likeness and Medicinal Chemistry Friendliness of Small Molecules. Sci. Rep..

[27] Molinspiration Cheminformatics 2006. https://www.molinspiration.com.

[28] Santafe G., Inza I., Lozano J.A. (2015). Dealing with the Evaluation of Supervised Classification Algorithms. Artif. Intell. Rev..

[29] Rainio O., Teuho J., Klén R. (2024). Evaluation Metrics and Statistical Tests for Machine Learning. Sci. Rep..

[30] Geng S. (2024). Analysis of the Different Statistical Metrics in Machine Learning. Highlights Sci. Eng. Technol..

